# Patient organisations and the reimbursement process for medicines: an exploratory study in eight European countries

**DOI:** 10.1186/1472-6963-10-45

**Published:** 2010-02-22

**Authors:** Janneke Noordman, Liset van Dijk, Roland Friele

**Affiliations:** 1 NIVEL, Netherlands Institute for Health Services Research, PO Box 1568, 3500 BN Utrecht, the Netherlands

## Abstract

**Background:**

Little is known about the role European patient organisations play in the process of deciding on reimbursement for medicines. Therefore we *explore *the current role of patient organisations in the process of reimbursement for medicines in Western Europe. We focus in particular on collaboration between patient organisations and the pharmaceutical industry in this respect.

**Methods:**

Sixty-eight patient organisations representing seven medical conditions, from ten Western European countries, were asked to participate in the study. The participating organisations reported their experiences in a web-based questionnaire.

**Results:**

Twenty-one patient organisations completed the questionnaire (response rate: 31%), of which ten (47.6%) demanded reimbursement for medicines. Organisations demanding reimbursement were larger than those not demanding reimbursement. The main aim of these organisations was to create better accessibility of medicines for patients. Most organisations limited themselves to single actions. Only two engaged in multiple actions. Almost all organisations had general policies on cooperation with the pharmaceutical industry, with autonomy as the key feature. The patient organisations said they were reasonably successful and almost always satisfied with their own role in the reimbursement process.

**Conclusion:**

Our study has found that the role of European patient organisations in the reimbursement process still seems limited, especially for small patient organisations.

## Background

Often, advanced and expensive medicines are not fully reimbursed. Stakeholders such as patient organisations may call for better reimbursement conditions [1,2]. The decision-making process regarding reimbursement differs between European countries. Differences also exist in the possibilities available to patient organisations to influence these decisions, depending on the reimbursement system of the country, the position of the patient organisation and the level of professionalization within the organisation [3]. Consequently, there are variations in reimbursement for medication between European countries. The Alzheimer associations, for example, state that access by European citizens to existing anti-dementia drugs is unequal. While in some countries, like Ireland and Sweden, all Alzheimer medicines are reimbursed without restrictions, other countries, such as the Netherlands and the UK exclude some medicines or treatment groups [4].

Patients and their representatives (e.g. patient organisations) are becoming increasingly important in creating access to healthcare and medicines. Patient organisations may act as an advocate for the patients they represent in the decision-making process regarding reimbursement for medicines. Since the authorities do not provide enough financial support for patient organisations to perform their activities, many organisations receive funding from the pharmaceutical industry [5,6]. The funding of patient organisations (and their relationship with the pharmaceutical industry) is frequently the subject of debate [5-13]. However, little is known about the role patient organisations occupy concerning the reimbursement of medicines. This lack of knowledge was reason for a group of patient organisations to ask NIVEL for an exploratory study in this field, in order to learn from other organisations' experiences. The aim of the present paper is therefore *to explore *the current role of patient organisations in the process of reimbursement of medicines in Western Europe. We focus in particular on the cooperation between patient organisations and the pharmaceutical industry, regarding the process of reimbursement for medicines. This collaboration with the industry remains complex, since conflicts of interest can appear when patient organisations do not maintain their independence [7,8]. The autonomy of patient organisations is jeopardised by the fact that they would be unable to function without financial aid from the industry. For instance, there is a risk that patient organisations could be used to sell the medicines supported by the industry [9]. At the same time, patient organisations and the industry share a common interest in creating access to specific treatments and medicines.

In sum, this paper's research questions are:

1. What is the role of European patient organisations in the process of reimbursement for medicines?

2. How successful are patient organisations in achieving their goals within this process?

3. How do patient organisations define their relationship vis à vis the pharmaceutical industry, regarding the reimbursement process of medicines?

The research was carried out by NIVEL (Netherlands Institute for Health Services Research) at the request of a steering committee, consisting of several Dutch patient organisations, and was funded by Glaxo Smith Kline (GSK). The steering committee of the 2007 meeting consisted of representatives from the Dutch Cancer Federation, the Netherlands Patient and Consumer Federation, Diabetes Union Netherlands and the Asthma Foundation. The committee was chaired by a former Dutch minister of Public Health (Borst). GSK did not participate in the design and reporting of the study, while the steering committee had an advising role in the design and reporting of the study. Confidentially was no issue, because the NIVEL-institute has the statutory obligation to publicly publish the results of its work. Furthermore, in the research contract independence and freedom of publication was secured.

## Methods

Sixty-eight patient organisations in ten European countries were asked to fill in a web-based questionnaire. The countries included were: Belgium, Denmark, Germany, Finland, France, Ireland, the Netherlands, the United Kingdom, Sweden, and Switzerland. These countries were chosen because they have a similar 'medicine culture', in contrast to for example southern European countries [14] and therefore more learning experiences for the Dutch situation could be expected. Moreover, language was an issue. The questionnaire was developed in four languages (Dutch, English, French and German) and from the selected countries we could expect that the representatives could read and understand one of these four languages.

For each country a maximum of seven organisations was contacted. We chose to contact four large categorical patient organisations because we hypothesized that to undertake actions a certain volume may be necessary and because these organisations may have more power in terms of money and personnel to influence reimbursement processes. The following four diseases were selected: diabetes, cardio-vascular disease, asthma (and allergy), multiple sclerosis. Since innovative medicines are developed for rare diseases, we decided to select a patient organisation for a rare disease as well. The choice for Addison Cushing was based upon a discussion with a senior researcher who knows the field of rare diseases. Finally two patient organisations were chosen for recently registered medication. Selection was based upon information of the websites of EMEA http://www.ema.europa.eu, the Dutch Medication Evaluation Board and through websites of patient organisations. In the end, Parkinson's disease and ADHD were selected.

Through searching the internet we obtained the addresses of the patient organisations we wanted to invite. Patient organisations were approached by e-mail and asked whether or not they wanted to participate in the study. In addition, we asked who was most eligible to fill out the questionnaire. We explicitly mentioned that the study was subsidized by GSK and that a steering committee of patient organisations had asked us to perform the study. The mail was sent out in Dutch, English, French or German. Respondents were asked to fill out a web-based questionnaire. After agreement upon participation the person who was mentioned as the respondent received an e-mail with a link to this questionnaire. The questionnaire was available for a one-month period. In order to increase the response rate all non-responding organisations were reminded three times. We considered three reminders to be still acceptable, without becoming too harassing.

We developed the questionnaire based on literature about patient organisations and the reimbursement process and on advices of members of the steering committee, existing of representatives of patient organisations. Since our study was the first specifically on this subject, it was hard to construct structured questionnaires with relevant answer categories. Therefore, we decided to use a questionnaire with open-ended questions. The questionnaire was reviewed by a number of fellow researchers with relevant experience as well as by a representative of a Dutch patient organisation with an interest in research. It included items on the following subjects: characteristics of the organisation, actions taken to obtain reimbursement, cooperation with other organisations and parties, evaluating the role of the organisation, recommendations for other patient organisations, and the policy of the organisation. If the organisations did not take action for the reimbursement process of a medicine, they were asked if they had ever considered doing so, whether their cooperation was requested by other organisations and why action was not taken (see table 1 for the research questions). To avoid bias in summarizing and reporting the results of the questionnaire all authors looked at the outcomes independently; JN summarized the findings, LvD and RF separately controlled the findings and reported results. Ethical approval for this type of research is not necessary according to Dutch law. Analyses were descriptive. We distinguished between patient organisations that took action for the reimbursement of medicines and those that did not.

**Table 1:** Questions in the on-line questionnaire

- Has your organisation ever taken action about the pricing/and or the payment allowance for a drug? *(e.g. by contacting other organisations or groups, submitting complaints, taking legal steps)*
- In the case of which drug(s) did your organisation take action?
- For which reason(s) did your organisation take action?
- What form did the action(s) take? *(please also refer to the authorities or bodies at which the action was aimed)*
- What did your organisation wish to achieve with the action?
- Did your organisation achieve its goal?
- Did your organisation take action alone or jointly with others?
- Which action(s) did your organisation undertake with others?
- With whom did your organisation undertake the action(s)?
- Who took the initiative to act jointly?
- With hindsight, how does your organisation evaluate its role in the process (according to level of satisfaction)? Please give your reasons for the answer.
- Which recommendations would your organisation give to other patient organisations? *(please indicate what you would advise and what you would advise against)*
- Does your organisation have a policy (protocol) on collaboration with the pharmaceutical industry? Please indicate the key points of your organisation's policy (or protocol).
*So far your organisation has taken ****no ****action in respect of the pricing or payment allowance for drugs. In that case please answer the questions below*.
- Has your organisation ever been approached with a view to taking action in respect of pricing and/or the payment allowance for drugs?
- Has your organisation ever considered taking action?
- By whom was your organisation approached?
- Why did you not take this up/take any action?

## Results

Representatives of twenty-one patient organisations, representing seven medical conditions and eight countries, reported their experiences in the web-based questionnaire (response rate: 31%). Two other patient organisations did not complete the questionnaire. No German or British patient organisations participated in the study. Belgium (n = 5) and the Netherlands (n = 4) had the highest response (see Table 2, 3).

**Table 2:** Patient organisations that participated in the study

*Responding patient organisations:*
- Vlaamse Vereniging van Cushing Addison, Belgium
- Astma en Allergiekoepel, Belgium
- Vlaamse Diabetes Vereniging, Belgium
- Nationale Belgische Multiple Sclerose Liga, Belgium
- Belgian Heart League, Belgium
- Dansk Parkinsonforening, Denmark
- Heart Foundation, Denmark
- Finnish Heart Association, Finland
- Allergia-ja Astmaliitto, Finland
- HyperSupers TDAH (ADHD), France
- Diabetes Federation, Ireland
- Young Parkinson, Ireland
- Asthma Society, Ireland
- Parkinson Patiënten Vereniging, Netherlands
- Stichting Hoofd Hart en Vaten (SHHV), Netherlands
- MS Vereniging, Netherlands
- Nederlandse Vereniging voor Addison en Cushing patiënten, Netherlands
- Astma och Allergi Förbundet, Sweden
- Swedish Diabetes Association, Sweden
- Riksförbundet Attention, Sweden
- Schweizerische Herzstiftung, Switzerland

**Table 3:** Patient organisations that did not respond to the request to participate in the study

*Non-responding patient organisations:*
- Centrum ZitStil (ADHD), Belgium
- Association Parkinson, Belgium
- Diabetesforeningen, Denmark
- Asthma and Allergy Forbundet, Denmark
- Scleroseforeningen, Denmark
- ADHD-foreningen, Denmark
- Addison Foreningen, Denmark
- Diabetesliitto, Finland
- Suomen MS-liitto ry, Finland
- ADHD liitto ry, Finland
- Suomen Parkinson-liitto ry, Finland
- Addison Support group, Finland
- French Federation of Cardiology, France
- Association Française des Diabétiques, France
- Association Asthme & Allergies, France
- Lique Francaise contre la Sclérose en Plaques, France
- Association France Parkinson, France
- Association Surrenales, France
- Herzstiftung, Germany
- Deutsche Diabetes-Union, Germany
- German Allergy and Asthma Association (DAAP), Germany
- Deutsche Multiple Sklerose Gesellschaft (DMSG), Germany
- Bundesverband Arbeitskreis Überaktives Kind, Germany
- PARKINSonLINE e.v. (PAoL), Germany
- Netzwerk Hypophysen- und Nebennierenerkrankungen, Germany
- Irish Heart Foundation, Ireland
- Multiple Sclerose Society (MSI), Ireland
- The North Fingal ADD/ADHD Parent and Adult Support Group, Ireland
- Diabetesvereniging, Netherlands
- Coalitie of Astmafonds, Vereniging Nederlands Davos & Astmapatiënten, Netherlands
- Balans & Impuls, Netherlands
- Heart and Lung Association, Sweden
- Neurologiskt Handikappades Riksforbund (NHR), Sweden
- Parkinson Förbundet, Sweden
- Stödföreningen Hypophysis, Sweden
- Swiss Diabetes Society, Switzerland
- Coalition of aha! Zentrum für Allergie, Haut und Asthma, Switzerland
- Schweizerische Multiple Sklerose Gesellschaft (SMSG), Switzerland
- Interessengruppe Aufmerksamkeitsdefizit-Syndrom, Switzerland
- Parkinson Schweiz, Switzerland
- British Heart Foundation, United Kingdom
- Diabetes UK, United Kingdom
- Asthma UK, United Kingdom
- MS Society, United Kingdom
- Thanet ADDers ADD/ADHD Support Group, United Kingdom
- Parkinson's Disease Society, United Kingdom
- Addison's Disease Self Help Group, United Kingdom/Ireland

### The role of patient organisations in the reimbursement process and their success

Eleven, mainly small-scale, independent organisations did not take action for the reimbursement of medicines (see table 4 for their characteristics). These organisations never considered starting a reimbursement process and did not cooperate with the pharmaceutical industry. Moreover, they were not asked by others to participate in actions to obtain reimbursement. Reasons for not taking action for the reimbursement of medicines were not given.

**Table 4:** Comparison between patient organisations that either did or did not try to obtain reimbursement

	Organisations that tried to obtain reimbursement (N = 10)	Organisations that did not try to obtain reimbursement (N = 11)
*Patient organisation* *(and number if >1)*	Asthma (and Allergy) Heart and vascular disease (3) Parkinson disease Diabetes (2) ADHD Multiple Scleroses	Asthma (and Allergy) (2) Heart and vascular disease (2) Parkinson disease (2) Diabetes ADHD Multiple Scleroses Addison and Cushing disease (2)
*Countries*	Belgium, Denmark, Ireland, Finland, Netherlands, Sweden	Belgium, Denmark, Ireland, France, Netherlands, Sweden Switzerland
*Average number of paid employees*	26 (range: 7-80)	5 (range: 0-17)
*Average number of volunteers*	200-500 (range: <200 - >500)	< 200 (range: <200-500)
*Average number of affiliated patients*	31.080 (range: 5.500 - 90.000)	9.805 (range: 850 -36.000)
*Sort organisation* *(and number if > 1)*^1^	Independent: 9 An umbrella for a number of smaller patient organisations: 2 Part of a European network of patient organisations: 6 Part of an international network of patient organisations: 6	Independent: 10 An umbrella for a number of smaller patient organisations: 2 Part of a European network of patient organisations: 6 Part of an international network of patient organisations: 2

^1 ^There are more answers possible for one organisation. For example, an organisation can be part of a European and international network.

Ten large-scale organisations demanded reimbursement. A number of these were part of a European and international network. A network consisted for example of Heart and vascular disease patient organisations in seven different countries. The ten organisations represented several medical conditions. Reimbursement was requested for a variety of drugs, such as statins, clopidogrel, asthma-inhalers and methylphenidate. The initiative to demand reimbursement always stemmed from the organisations themselves. Respondents reported that the pharmaceutical industry did not approach the patient organisations to take action regarding reimbursement of medicines. Patient organisations cited the following reasons for requesting reimbursement: to create access to a medicine, to seek agreement on reimbursement of a higher dose, to facilitate proper treatment and to reimburse a medicine that is frequently prescribed by doctors. Most of these organisations limited themselves to single activities: writing one or more letters or participating in a consultation session. Two organisations (in Sweden and the Netherlands) took multiple actions. They wrote letters, invited the concerned parties for a consultation session, contacted the media and funded research on the living conditions of members (see Table 5). Half of the organisations cooperated with others, for example with other patient organisations, individual patients, medical specialists or nurses and/or the pharmaceutical industry. Patient organisations evaluated their actions as reasonably successful and were almost always satisfied with them. We asked the patient organisations that took action whether they had recommendations for other organisations and if so what they would advise. The results were ambiguous: some organisations recommended cooperating with professionals and their organisations while others advised against depending on other organisations. Solid information facilities and a professional management for patient organisations were mentioned several times.

**Table 5:** Medication for which reimbursement was sought and actions of the patient organisation

*Medication*^2^	*Patient organisation*	*Actions*
Atorvastatin and rosuvastatin	Heart Association, Finland	(Special) reimbursement application directed to the concerned authorities
Statins	Diabetes Federation, Ireland	- Lobbying the government (national and local level) for complete reimbursement. The Departement of Health and politicians were asked to support the application
Methylfenidate	Riksförbundet Attention, Sweden	- Informing the media - Broadcasting the names of individuals who needed the medication - Commissioning university research on the life conditions of members.
Glargin and detemir	Vlaamse Diabetes Vereniging, Belgium	Providing scientific advice to the concerned Minister.
Asthma inhaling medication	Astma och Allergi Förbundet, Sweden	Letters to the concerned authorities.
Clopidrogel	Stichting Hoofd Hart en Vaten (SHHV), Netherlands	- Inviting the concerned parties around the table - Informing the media - Letters to the concerned authorities
Statins	Heart Foundation, Denmark	Letters to the concerned authorities.
Interferon-beta	Nationale Multiple Sclerose Liga, Belgium	Written protest to the Department of Health.
Combination of levodopa&carbidopa (duodopa)	Parkinson Association, Belgium	Letters to the concerned authorities.
Rasagilin	Parkinson Patiënten Vereniging, Netherlands	Letters to the concerned authorities.

^2 ^We asked for the most recent medicine for which action was taken (January 2007). Some patient organisations have taken more actions than for the above mentioned medicines, but they did not specify their actions for these medicines.

### Policy and cooperation with the pharmaceutical industry

We looked at how patient organisations define their relationship with the pharmaceutical industry regarding the reimbursement of medicines, based on their policy.

Almost all patient organisations in our study had a policy of (potential) cooperation with the pharmaceutical industry. Usually, this policy was formulated in general terms, with a strong emphasis on independence and openness. Large, professionalized organisations had more detailed policies, sometimes aimed at cooperation with the industry. Consequently, patient organisations that requested reimbursement had more detailed policies. Five small-scale patient organisations, that did not start a reimbursement procedure, had no policy at all. Reasons for lacking a policy were not provided.

## Discussion

This exploratory, small-scale study shows that the role of European patient organisations in the reimbursement process of medicines for the seven conditions we studied was generally limited. Still, some patient organisations adopted a role in this process.

In general, only the larger patient organisations played a part in the decision-making process regarding compensation for medicines. Their main aim was to acquire better accessibility of medicines for patients. They called for total reimbursement for everyone or for an enlargement of the patient groups for whom the medicine is reimbursed. They lobbied regarding a variety of medicines and conditions. Most organisations restricted themselves to simple, one-off actions. Only a few engaged in multiple actions. This study also showed that all the patient organisations that adopted a role in the reimbursement process did have policies on how to cooperate with the pharmaceutical industry. The fact that large patient organisations were more likely to take action in relation to the reimbursement process implies that a certain degree of organisation or professionalization is necessary to take up any role. The influence of the patient organisations might be more widespread if they were to collaborate to achieve their aim. Patient organisations seem not to be unaware of the potential influence of the pharmaceutical industry on their autonomy, by explicitly stating their policies. However, these policies were usually stated in general terms. It remains to be seen whether such general terms provide enough clarity to effectively sustain the independence of patient organisations. Besides, previous research indicates that only 18 percent of Dutch patient organisations have an official policy for cooperation with the pharmaceutical industry [13]. Many patient organisations (all across Europe) do not disclose clear information about their funding or competing interests [5,9]. According to the Dutch 'code of conduct for sponsorship' patient organisations should describe the sponsored activities in detail (with full financial disclosure) as well as the rights and obligations of all involved parties [15].

It should be noted that this study was exploratory, and only twenty-one patient organisations participated (response 31%). For two countries, England and Germany, no respondents were found. In Germany, patient organisations have no formal role in the reimbursement process, but in the United Kingdom they have [16]. Reasons for not participating (and withdrawal from the research) were not given. The reasons may have to do with a lack of time, a negative attitude towards sponsorship by the pharmaceutical industry or the absence of any activities in this domain. Besides, responses to web-based questionnaires are in general lower than those to mail questionnaires [17-19]. Moreover, we had to rely on self-reported answers from one representative of the patient organisation. The limited size of this study also restricted the depth of our analyses. Differences between countries could not be studied. In addition, we only included a selection of patient organisations. Although we reassured to have a range of different diseases, it is not known whether these organisations are representative for all diseases. Because of the explorative character of this study, the low response rate and the selection of patient organisations our findings cannot be generalized and further research is needed. This is all the more important because patient organisations will become a more powerful force, and will aim to influence decisions on budget-allocation in health care. The reimbursement process offers promising opportunities for studying this development. A larger scale study could provide insight into the role of patient organisations in different countries. The potentially multi-stranded relationship of patient organisations with the pharmaceutical industry, possibly leading to a loss of autonomy, should be considered in such a study.

## Conclusion

The role of patient organisations in the process of obtaining reimbursement for medicines is varied but seems limited. Only large and more professional organisations seem to play an active role in this respect. Nevertheless, it should be stressed that patient organisations have the potential to play an important part in the reimbursement process, i.e. contribute to the debate about reimbursement of medicines. As a result, organisations should be aware of the importance of stating an official, detailed and transparent policy regarding (cooperation with) the pharmaceutical industry.

## Competing interests

The research was funded by Glaxo Smith Kline (GSK). GSK played no role in the design and reporting of the study. The steering committee had an advising role in the design and reporting of the study. The authors declare that they have no other competing interests.

## Authors' contributions

RF conceived and supervised the study and helped to draft the manuscript. LvD co-ordinated the study and helped to draft the manuscript. JN carried out the study and drafted the manuscript. All authors read and approved the final manuscript.

## Pre-publication history

The pre-publication history for this paper can be accessed here:

http://www.biomedcentral.com/1472-6963/10/45/prepub

## References

[B1] Koning J De Besparen op medicijnen [Saving on medicines] Patiënt en Perspectief 1998 5 17 22

[B2] Koning J De Doorzakken is geen ziekte [In sagging is no sickness] Patiënt en Perspectief 1998 5 26 28

[B3] Jack A Too close for comfort? Financial Times, British Medical Journal 2006 333 13

[B4] Georges J The Availability of Antidementia Drugs in Europe Alzheimer Europe/European Neurological disease 2007

[B5] Herxheimer A Relationships between the pharmaceutical industry and patients' organisations British Medical Journal 2003 326 1208 1210 10.1136/bmj.326.7400.1208 12775627 PMC1126060

[B6] Vermeulen M Bouma J Invloed van de farmaceutische industrie in patiëntenverenigingen [The influence of the pharmaceutical industry in patient organisations] Nederlands Tijdschrift voor Geneeskunde 2007 151 41 2432 2434 18064861

[B7] Tuffs A Sponsorship of patients' groups by drug companies should be made transparent British Medical Journal 2006 333 1238 10.1136/bmj.39062.360289.DB 17170403 PMC1702432

[B8] Burton B Drug companies told that sponsoring patients' groups might help win approval for their products British Medical Journal 2005 331 1359 10.1136/bmj.331.7529.1359-b 16339237 PMC1309681

[B9] Ball DE Tisocki K Herxheimer A Advertising and disclosure of funding on patient organisation websites: a cross-sectional survey BioMed Central Public Health 2006 16887025 10.1186/1471-2458-6-201 PMC1557495

[B10] Ternhag A Asikainen T Giesecke J Size matters - patient organisations exaggerate prevalence numbers European Journal of Epidemiology 2005 20 653 655 10.1007/s10654-005-0705-8 16151877

[B11] Mintzes B Should patient groups accept money from drug companies? No British Medical Journal 2007 334 935 10.1136/bmj.39185.394005.AD 17478846 PMC1865416

[B12] Kent A Should patient groups accept money from drug companies? Yes British Medical Journal 2007 334 934 10.1136/bmj.39185.461968.AD 17478845 PMC1865450

[B13] Rijn van Alkemade EMv Sponsoring van patiëntenorganisaties door de industrie [Sponsorship of patient organisations by the industry] DGV, Nederlands instituut voor verantwoord medicijngebruik 2005

[B14] Kooiker S Wijst L Van der Europeans and their medicines. A cultural approach to the utilization of pharmaceuticals Den Haag. SCP/GfK 2002

[B15] CGR Gedragsregels Sponsoring [code of conduct for sponsorship] 2008 http://www.cgr.nl

[B16] Noordman J Van Dijk L Friele R Een nieuw medicijn: hoe Europese patiëntenorganisaties een rol kunnen spelen bij het beschikbaar komen van nieuwe medicijnen [A new medicine: how European patient organisations can play a role in the process of reimbursement for medicines] Utrecht Nivel 2007

[B17] Kongsved SM Basnov M Holm-Christensen K Hjollund NH Response rate and completeness of questionnaires: a randomized study of internet versus paper-and-pencil versions J Med Internet Res 2007 9 3 10.2196/jmir.9.3.e25 17942387 PMC2047288

[B18] Van Selm M Jankowski NW Conducting online surveys Quality & Quantity 2006 40 435 456

[B19] Leece P Bhandari M Sprague S Swiontkowski MF Schemitsch EH Tornetta P Devereaux PJ Guyatt GH Internet versus mailed questionnaires: a controlled comparison (2) J Med Internet Res 2004 6 4 10.2196/jmir.6.4.e39 PMC1550620 15631963

